# Trace Determination of Cyanide in Chinese Liquor by Coupling Automatic Distillation with the Flow Analysis Method

**DOI:** 10.3390/foods14173045

**Published:** 2025-08-29

**Authors:** Lingyi Zhao, Youquan Zhao, Lvbo Wang, Junjie Zhang, Yanjun Fang

**Affiliations:** 1 Academy of Medical Engineering and Translational Medicine, Tianjin University, Tianjin 300072, China; zhao_01@tju.edu.cn (L.Z.); wanglvbo2022@163.com (L.W.); 18778938320@163.com (J.Z.); 2 The Key Laboratory of Risk Assessment and Control Technology for Environment and Food Safety, Tianjin Institute of Environmental and Operational Medicine, Tianjin 300050, China; fangyj86@126.com

**Keywords:** cyanide determination, distillation, continuous flow analysis, food safety

## Abstract

Cyanide, a highly toxic compound, may exist in trace amounts in distilled Chinese liquor, a traditional spirit consumed by over a billion people. To enhance the efficiency and reliability of cyanide detection, we propose a rapid, fully automated method integrating steam distillation with a continuous flow analyzer. This system combines sample pretreatment, fast distillation, and colorimetric detection using the isonicotinic-barbituric acid chromogenic system based on Lambert–Beer law (measuring at 600 nm). The standard curve exhibits an excellent linear relationship and a correlation coefficient r = 0.99959. The detection limit (0.2519 µg/L) was derived from 11 replicate blank measurements. When applied to three types of Chinese liquor, the method yields spike recovery rates ranging from 91.530% to 98.485%, and precision values between 1.177% and 1.525%. A paired *t*-test confirms no significant difference between this method and the national standard spectrophotometric method (*t* = 0.535, *p* = 0.615). Overall, this method offers outstanding repeatability, high accuracy, rapid analysis, and minimal manual intervention, establishing it as a reliable tool for cyanide determination and contributing to the assurance of food safety in alcoholic beverages.

## 1. Introduction

Chinese liquor, one of the world’s six most famous distilled spirits, has a long production history. It is distinguished by the use of specially designed fermentation starters [1], solid-state fermentation, and distillation in traditional Chinese stills [2]. Chinese liquor is becoming a high-value commercial product, favored by more than a billion people. Cereals with naturally high sugar content or easily convertible starches, such as sorghum, rice, wheat, corn, glutinous rice, and barley, are commonly used as raw materials for Chinese liquor production [3]. However, cyanide is often found in these cereals. A trace amount of hydrogen cyanide has also been detected in distilled liquors and other alcoholic beverages. Cyanogenic glycosides have been identified in over 2000 plant species. Cyanide is also a precursor of ethanol and contributes to the formation of ethyl carbamate (EC) during alcohol fermentation [4,5,6]. Cyanide is produced by the enzymatic or acid thermal decomposition of cyanoglycosides from grains and the metabolism of arginine during Lactobacilli fermentation. Ethyl carbamate in alcoholic fermentation is mainly generated by the reaction of urea and ethanol. In the early stage of Chinese liquor distillation, steam brings a small part of ethyl carbamate from alcoholic fermentation. In the late stage, ethyl carbamate is mainly derived from the reaction between the ethanol and distillation precursors such as cyanide. Cyanide is also the main precursor for ethyl carbamate formation during the ripening process. The amount of cyanide has been shown to increase proportionally with the level of EC [7]. According to the International Agency for Research on Cancer [8], EC shows strong carcinogenic potential. Alcoholic beverages are considered the most important source of ethyl carbamate intake for most people. Thus, the content limitation of ethyl carbamate for distilled spirits was set at 150 μg/L in America, Brazil, and Canada, while it was not regulated in Chinese liquor due to the lack of relevant research and evaluation. Therefore, it is important to accurately determine trace cyanide content using analytical methods.

In practice, many factors are critical to ensuring the efficiency and accuracy of cyanide analysis. These include temperature, the type of distillation equipment used, and the time required to separate, extract, and detect cyanide from both cereal matrices and liquor.

Current methods for cyanide determination mainly rely on the spectrophotometric technique specified in GB 5009.36—2016, National Food Safety Standard for the Determination of Cyanide in Food. In addition, other commonly used techniques include chromatographic methods [9,10], continuous flow analysis, titration methods [11,12], and rapid detection techniques [13]. It is well known that conventional distillation procedures are complex, time-consuming, and inefficient. They often take over two hours per run and have low recovery rates, typically below 65%. Although Qin et al. [14] developed a method to simultaneously determine volatile phenols, cyanide, anionic surfactants, and ammonia nitrogen in drinking water using a continuous flow analyzer, its application in liquor is limited.

Similarly, Virbickas et al. [15] applied chronoamperometry, cyclic voltammetry, and fast Fourier transform electrochemical impedance spectroscopy to detect cyanide, but these methods still lacked high accuracy. La et al. [16] used fluorescent probes for detection. However, these probes suffered from poor solubility, a lack of biological specificity, and high sensitivity to environmental conditions such as temperature and pH, which significantly hindered their practical use. In recent years, non-destructive testing technology has also provided new ideas for cyanide analysis. For example, the Raman integrating sphere method developed by Xu et al. [17] achieved the in situ detection of cyanide in liquor (LOD = 1.56 mg/L), but it was significantly disturbed by the ethanol matrix (RSD up to 39%).

In contrast, the spectrophotometric method remains the most widely used. It is based on the Lambert–Beer law and the isonicotinic-barbituric acid system, which forms a stable blue-violet complex measurable at 600 nm. This system offers a short color development time, simple operation, and does not require special solvents, making it suitable for high-throughput analysis.

In this study, we developed an automated distillation system to minimize manual intervention and shorten cyanide recovery time. The ADFA method (automatic distillation-flow analysis method) achieves rapid detection (10 min/sample) using commercially available components (HGCF-100 analyzer). This aligns with Qin’s vision of simplified automation but specifically optimized for liquor. Unlike Raman spectroscopy, which suffers 39% RSD in a high-concentration ethanol matrix, our distillation step eliminates ethanol interference, achieving RSD < 1.53% in the liquor sample. This solves the key limitation reported by Xu et al. [17] for in situ liquor analysis. The novelty of ADFA lies not in the simple combination of established techniques, but in addressing critical challenges in cyanide detection in distilled spirits, creating a versatile and cost-effective approach that can be implemented with minimal setup and at lower operational costs. Furthermore, our method’s automation reduces the likelihood of human error and improves reproducibility, making it particularly useful for large-scale or field-based applications where time and accuracy are critical. By simplifying the procedure while maintaining high accuracy, we believe our system offers a unique advantage, particularly for applications where speed, reliability, and cost-efficiency are paramount.

Method validation included experiments on calibration curves, accuracy, and spike recovery. Parallel tests showed no significant difference between our method and the national standard. Overall, this study proposes a rapid, efficient, and high-recovery method for trace cyanide detection in Chinese liquor, supporting future applications in automated analytical instrumentation.

## 2. Materials and Methods

### 2.1. Samples and Reagents

Samples: The liquor purchased comes from different supermarkets in Tianjin, with three different aroma types, and six parallel experiments were designed. Then randomly number the samples of each fragrance type (1–6).

To minimize subjective bias during method validation, a blinding protocol was implemented. Sample blinding: All liquor samples were labeled with random codes by an independent researcher. Spike blinding: Concentrations of cyanide spikes were prepared by a separate technician and concealed from analysts. Analysis blinding: Operators performing distillation, flow analysis, and data recording were sequentially segregated and unaware of sample identities or expected outcomes.

Decoding occurred only after statistical completion.

Cyanide standard solution (GBW(E)080115, 50 mg/L, KCN in 0.1 mg/L NaOH) was purchased from the National Institute of Metrology of China. Chloramine T was purchased from Damao (Tianjin, China). Isonicotinic acid was purchased from Jinke (Tianjin, China). Zinc acetate, sodium hydroxide, potassium hydrogen phthalate, potassium hydroxide, 1,3-dimethyl barbituric acid, ethanol, phenolphthalein, tartaric acid, pyrazolone, acetic acid, anhydrous potassium dihydrogen phosphate, and anhydrous sodium dihydrogen phosphate were purchased from Sinopharm Chemical Reagent Co., Ltd. (Shanghai, China). The liquor samples analysed were purchased from different supermarkets in Tianjin, including three types: sauce-flavour, strong-flavour, and light-flavour. All the reagents used were analytical grade and above.

Standard solution: Pipette 0.1, 0.2, 0.4, 0.6, 0.8, 1.0, and 1.2 mL of the cyanide standard intermediate solution (1.0 μg/mL) into 10 mL stoppered colorimetric tubes and make up to 10 mL with sodium hydroxide solution (0.01 mol/L) to obtain the prepared cyanide standard working solutions with mass concentrations of 10, 20, 40, 60, 80, 100, and 120 μg/L.

Buffer solution: Dissolve 9.2 g of sodium hydroxide and 82 g of potassium hydrogen phthalate in 800 mL of water, make up to 1 L with water, mix well, and adjust the pH to 5.2 ± 0.1.

Isonicotinic-barbituric acid solution: Dissolve 4.4 g of potassium hydroxide, 8.4 g of isonicotinic acid, and 6.8 g of 1,3-dimethyl barbituric acid in 300 mL of water, heat in a water bath, cool to room temperature, make up to 500 mL with water, mix well, and adjust the pH to 5.2 ± 0.1. The reagent is stable for up to one week at room temperature, and it is not recommended to refrigerate it to avoid crystallization.

Chloramine T solution (2 g/L): 1 g of chloramine T dissolved in water, and dilute to 500 mL, prepare immediately before use.

The preparation of reagents for the comparative experiment is carried out according to the spectrophotometry and gas chromatography requirements outlined in GB5009.36-2016 “National Food Safety Standard for the Determination of Cyanide in Food”.

### 2.2. Instrumentation

Automatic steam distillation instrument: independently developed by Tianjin University. Spectrophotometer (UV-2450): Shimadzu Corporation, Kyoto, Japan. Stirrer (SN-JJ): Shanghai Shangyi Co., Ltd., Shanghai, China. Cyanide continuous analyser (HGCF-100), injector (ASX-280): Beijing Haiguang Instrument Co., Ltd. The HGCF-100 cyanide continuous flow analyzer is a commercially available system. Daily calibrating with certified KCN standards (GBW(E)080115, 0–100 μg/L) before each use.

### 2.3. Establishment of Standard Curve and Data Analysis Method

**Figure 1:**
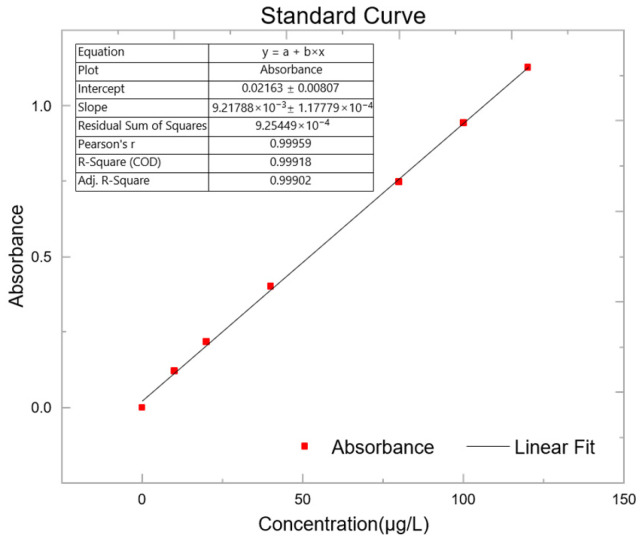
Standard curve. Prepare different standard solutions of cyanide, measure the absorbance respectively, and fit the standard curve based on the results.

The concentrations of the cyanide standard working solutions ranged from 0 μg/L to 120 μg/L. A continuous flow analyzer was used for the determination. A standard curve was constructed by plotting the cyanide concentration (μg/L) on the x-axis and the absorbance on the y-axis (Figure 1).

The linear regression equation of the standard curve is y = 0.00922x + 0.02163, with a correlation coefficient (r) of 0.99959, indicating excellent linearity. The method detection limit (MDL) was calculated by measuring a blank sample 11 times and taking three times the standard deviation of the measured values, yielding a detection limit of 0.2519 μg/L.

**Figure 2:**
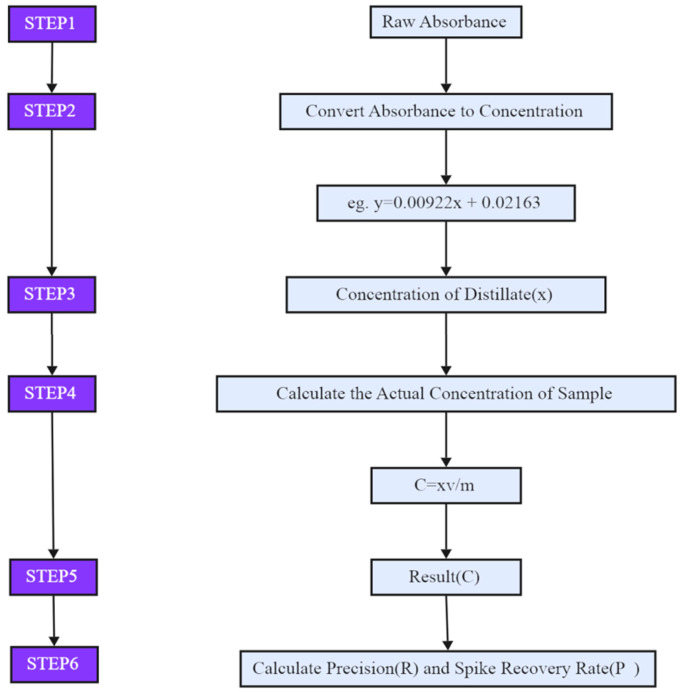
Traceability flow of calculation of results.

Data analysis was performed using Excel to calculate the cyanide content, precision, and spike recovery in food samples. Origin 2025b was used for graphing and conducting *t*-tests to compare the consistency between the two experimental methods. The specific calculation process is shown in Figure 2.

The formulas used are as follows:

C = xv/m(1)

where 

C: cyanide content in the sample (as CN^−^) (mg/L);

x: cyanide concentration read from the standard curve (μg/L);

v: volume of distillate (L);

m: mass of the sample taken (for solids: g, for liquids: mL).

R = S/A(2)

where

R: precision (%);

S: standard deviation (mg/kg or mg/L);

A: average value (mg/kg or mg/L).

P = (T − t_0_)/t (3)

where

P: spike recovery rate (%);

T: measured value (mg/L);

t_0_: background value (mg/L), and t denotes the Spiked amount (mg/L).

### 2.4. Fully Automatic Steam Distillation-Continuous Flow Analysis Method

Cyanide continuous flow analyzers are generally designed to detect only liquid samples. To expand the range of analyzable sample types, this study proposes combining an automatic steam distillation unit with the HGCF-100 cyanide continuous flow analyzer. This integration enables rapid extraction and detection of cyanide in food samples.

**Figure 3:**
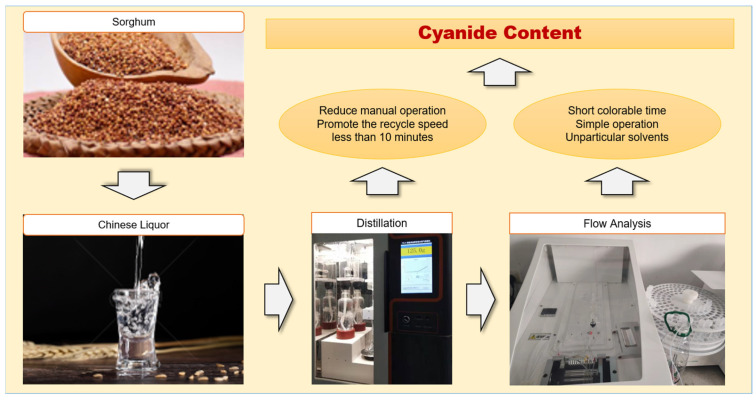
Schematic diagram of the ADFA method.

Steam distillation is widely used for extracting active compounds from samples. Due to the lack of dedicated distillation equipment for food safety testing, many current methods utilize a Kjeldahl nitrogen analyzer. However, this instrument is costly, complex to operate, and designed specifically for protein titration, making it unsuitable for cyanide detection, which does not require titration. To address this, we developed a specialized steam distillation apparatus under the ADFA method (Figure 3). This system includes a high-capacity digestion tube, an efficient waterless condenser, and a programmable endpoint control for quantitative analysis. It is specifically designed for cyanide extraction. While conventional sample preparation takes 1 to 2 h, our apparatus reduces this to approximately 10 min.

A rapid detection system was developed by integrating cyanide detection techniques with steam distillation and colorimetry. To minimize cyanide loss caused by heat during the grinding of solid samples, the samples were cryopreserved and ground into powder. Sodium hydroxide was used to stabilize the cyanide in the samples. Previous studies have shown that the distillation of hydrogen cyanide requires an acidic environment. In this study, a tartaric acid–zinc acetate system was used to create the required conditions. Tartaric acid first complexes with metal ions, reducing their ability to bind cyanide ions. As a common food antioxidant, it also mitigates interference from hypochlorite. Zinc acetate was added before distillation to eliminate interference from sulfide ions. Finally, cyanide was efficiently volatilized and separated under optimized distillation conditions.

The self-developed intelligent steam distillation system was used to extract cyanide from food samples. Subsequently, the upgraded HGCF-100 cyanide continuous flow analyzer (Beijing Haiguang Instrument) was employed. During operation, the distillate, buffer solution, chloramine T, isonicotinic-barbituric acid reagent, and air were sequentially introduced into the flow system. The analyzer uses bubble segmentation technology to minimize cross-sample interference, ensuring better reaction completeness and analytical stability.

To begin the process, the intelligent steam distiller is activated, and sodium hydroxide is added to the distillate receiving flask to stabilize the extracted cyanide. A defined amount of food sample is weighed, combined with reagents to establish an acidic environment, and transferred into the distillation reaction flask. Users can pre-set the target distillate volume. Once the specified volume is reached, the device shuts off automatically. The distillate is then transferred to a 50 mL stoppered colorimetric tube and diluted to volume using 0.01 mol/L sodium hydroxide.

The resulting solution is introduced into the auto-sampling system of the continuous flow analyzer, where it reacts with the isonicotinic-barbituric acid system. Absorbance is measured at 600 nm, and the cyanide concentration is quantified by comparison with the standard calibration curve.

This system features rapid heating, a distillation power of up to 2000 kW, customizable program control, and fully automated colorimetric detection. These enhancements significantly reduce analysis time, minimize human error, and improve both the speed and accuracy of cyanide quantification in food samples.

## 3. Results and Discussion

### 3.1. Optimization of the Heating Temperature of the Water Bath

Isonicotinic acid and barbituric acid are both soluble in hot water and under alkaline conditions. The heating temperature has a significant effect on the chromogenic reagent. At low temperatures, incomplete dissolution may occur, leading to precipitation. However, excessively high temperatures can alter the chemical properties of the reagents.

To determine the optimal temperature for reagent preparation, we examined the linear correlation coefficients of standard curves generated under different temperature conditions. The results are summarized in Table 1. Based on these results, 60 °C was selected for subsequent experiments, as it produced the highest linearity with a very small error in the standard curve, indicating a stable and reproducible relationship between temperature and the measured response. The high slope at 60 °C is important for the accuracy of the analytical method, and its slope is closer to those observed at other temperatures with steeper slopes. Moreover, the R^2^ value at 60 °C (0.99900) is significantly higher than at 80 °C (0.90640), which further demonstrates that the repeatability and reliability of the experiment are best at 60 °C.

### 3.2. Method Application

**Figure 4:**
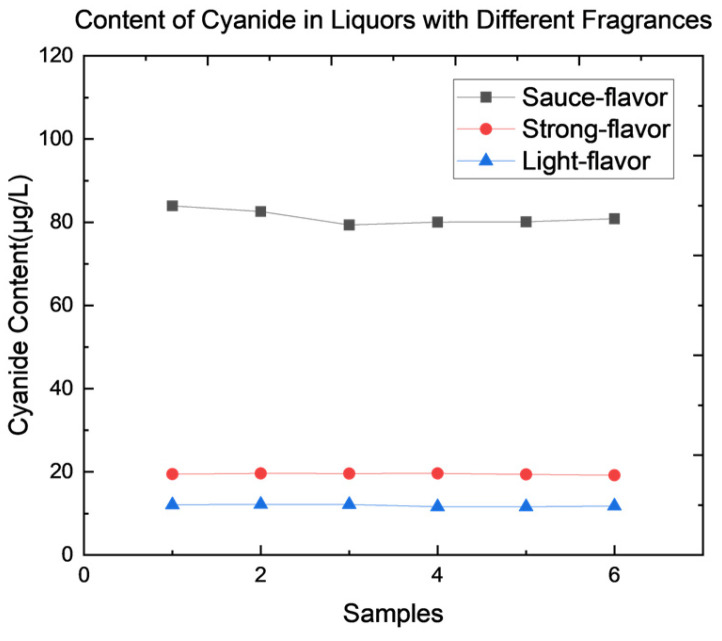
Content of cyanide in Chinese liquors with three different fragrances.

Three different fragrance types of Chinese liquor were selected, and parallel trials were conducted for each type (Figure 4). The cyanide content in all three types was within the national safety limit of 8.0 mg/L. However, significant variation was observed among the different types. The sauce-flavour Chinese liquor contained notably higher cyanide levels than the strong-flavour and light-flavour types. This difference is likely related to variations in the production process.

Sauce-flavour Chinese liquor is produced at fermentation temperatures exceeding 60 °C and undergoes multiple rounds of fermentation and distillation. Prior to fermentation in cellars, a stacking (piling) stage is carried out, with a storage duration of up to three years.

During this phase, the enzyme β-glucosidase repeatedly hydrolyzes cyanogenic glycosides into sugars and α-hydroxynitriles. These α-hydroxynitrile compounds can release hydrogen cyanide and aldehyde–ketone derivatives, either spontaneously or through the action of α-hydroxynitrile lyase. Additionally, some cyano-hydrins may undergo degradation during the stacking and fermentation stages, potentially releasing further cyanide species. During distillation, any cyanogenic glycosides that were not fully decomposed during fermentation can continue to break down, further increasing the free cyanide content.

Although these biochemical processes suggest a potential link between fermentation conditions (such as temperature) and cyanide formation, we currently lack direct compositional data (e.g., GC-MS or LC-MS analysis) to confirm the presence of cyanogenic glycosides or intermediates at different fermentation temperatures. Future studies will focus on analyzing these compounds to provide stronger evidence for the relationship between fermentation conditions and cyanide formation, which will help clarify the underlying mechanisms.

The Chinese National Standard outlines a method for determining cyanide content in distilled spirits. Under alkaline conditions, high-boiling organic compounds are removed by heating. Cyanide is then converted into hydrochloric acid using chloramine T at pH 7.0. The resulting hydrochloric acid reacts with isonicotinic acid–pyrazolone to produce a blue dye, the absorbance of which is measured at 638 nm and quantified against a standard calibration curve.

The heating step involves reducing the solution volume to approximately 1 mL using an electric heating plate at 120 °C. For turbid or colored samples, approximately 50 mL of distillate must be collected, which limits the accuracy of quantification. In addition, the standard method is not suitable for food samples containing amygdalin. Amygdalin decomposes into benzaldehyde and hydrogen cyanide, which enter the distillate. Benzaldehyde can react with pyrazolone to form a white, insoluble precipitate, interfering with absorbance measurements. Amygdalin is commonly found in the seeds of many fruits.

Given the complex composition of Chinese liquor, the ADFA method adopts the isonicotinic-barbituric acid colorimetric system to ensure accurate and reliable results. To evaluate the consistency between the ADFA method and the national standard method, a paired *t*-test was performed.

**Figure 5:**
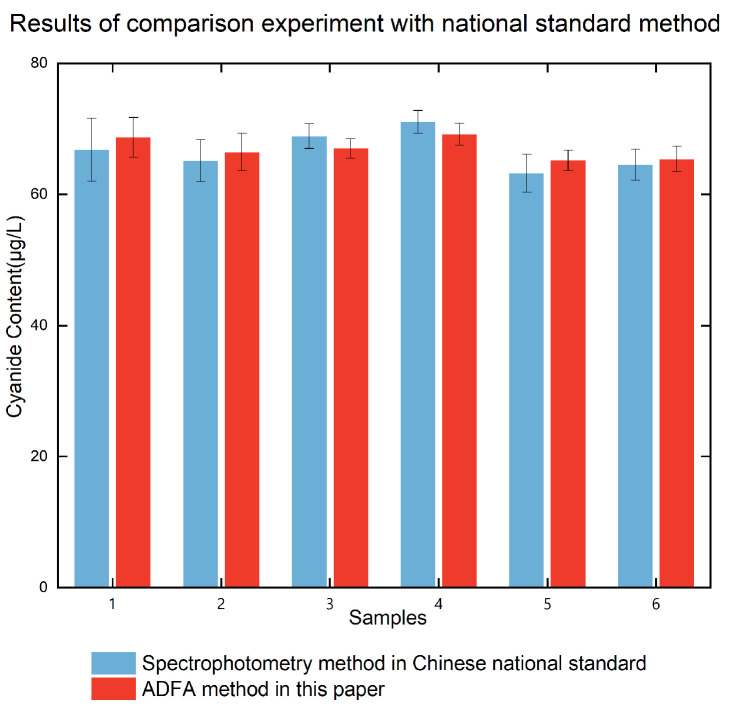
Results of the comparison experiment with the national standard method.

The *t*-test assesses whether the difference between the means of two paired samples is statistically significant. To verify our method, a paired *t*-test was conducted between ADFA with the national standard (Figure 5). The number of paired comparisons (n) used in the analysis was 6. The difference between methods had a mean of −0.4845 μg/L with a standard deviation of 1.546 μg/L. The 95% confidence interval (CI) for the difference between methods was [−2.1115, 1.1425] μg/L. The *t*-test yielded a *t*-value of 0.535, with a *p*-value of 0.615. Since the *p*-value exceeds the commonly accepted significance threshold (e.g., 0.05), we fail to reject the null hypothesis. This indicates that there is no statistically significant difference between the results obtained by the two methods.

**Figure 6:**
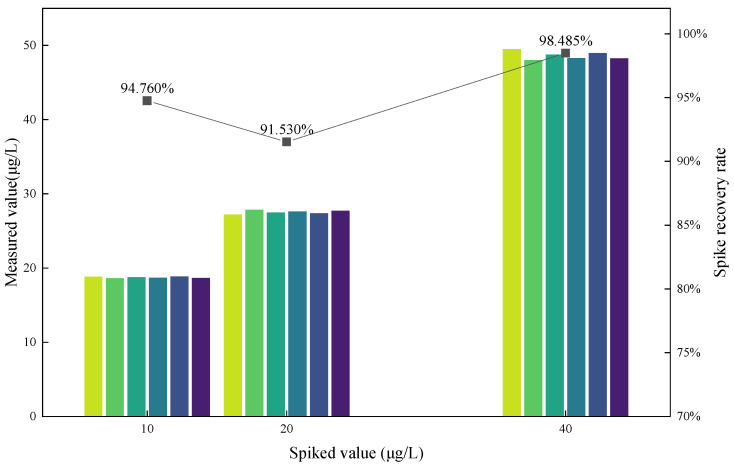
Graph of spike recovering experiment.

Cyanide standard solutions at concentrations of 10 μg/L, 20 μg/L, and 40 μg/L were added to the samples. Both the method proposed in this study and the spectrophotometric method specified in the Chinese National Standard were used to calculate spike recovery rates. The recovery rates for cyanide in the spiked samples ranged from 91.530% to 98.485%(Figure 6). To clarify, recovery values were calculated by subtracting blank values, but matrix-matched standards were not used in the current analysis. Therefore, matrix effects were not specifically evaluated or adjusted for in this study. The potential influence of matrix effects will be considered in future research, and matrix-matched standards will be employed to improve the reliability and accuracy of recovery calculations.

When comparing recovery performance, the method presented in this study showed superior results (Table 2). The national standard method involves concentrating 2 mL of solution on an electric heating plate at 120 °C. This heating and concentration step may cause significant cyanide loss, leading to lower recovery rates and reduced accuracy.

**Figure 7:**
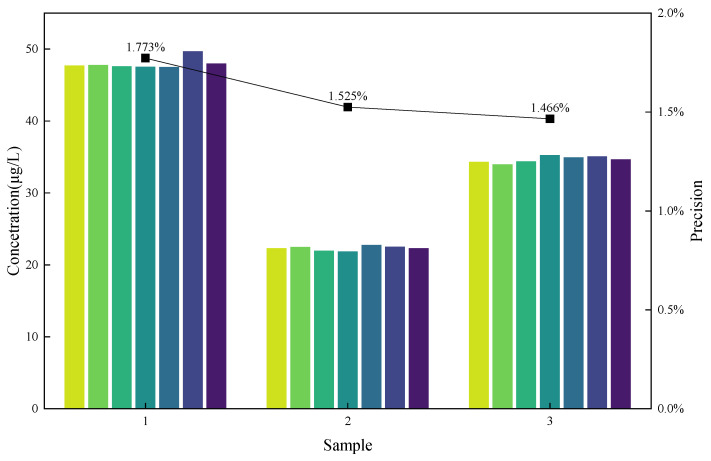
Graph of precision experiment.

According to the national standard, the absolute difference between two independent measurements under repeatability conditions must not exceed 10% of their arithmetic mean. Based on this requirement, precision experiments were conducted by analyzing each sample six times(Figure 7). The results are shown in Table 3. The measured precision ranged from 1.177% to 1.525%, all below the 10% threshold, indicating good repeatability and high method reliability.

### 3.3. Scope and Limitations

While this study validated the ADFA method for clear Chinese liquors, its performance in highly turbid or pigmented matrices requires further investigation. Colorimetric detection may face challenges in such complex systems due to spectral interference from anthocyanins/caramel colorants and particulate-induced light scattering during flow analysis.

Distillation partially mitigates these issues by separating cyanide from non-volatile interferents, but matrix-specific optimization would be needed for solid-rich samples. Future work will extend validation to broader food matrices, leveraging the system’s modular design.

## 4. Conclusions

This study presents a novel automated method integrating steam distillation with continuous flow analysis for trace cyanide detection in Chinese liquor. The method demonstrates exceptional analytical performance, including a detection limit of 0.2519 μg/L (calculated from 11 blank replicates), linearity with r = 0.99959, and spike recovery rates ranging from 91.53% to 98.48% across three Chinese liquor fragrance types. Precision values (<1.525%) confirm high repeatability, while a paired *t*-test (*t* = 0.535, *p* = 0.615) validates consistency with the national standard method. By reducing analysis time from 2 h to under 10 min and minimizing manual intervention, this approach addresses key limitations of conventional techniques. The integration of automated sample pretreatment, fast distillation, and colorimetric detection based on the isonicotinic-barbituric acid system establishes a reliable, high-throughput solution for food safety monitoring. This method establishes a robust platform for cyanide monitoring in distilled spirits, with potential adaptability to other liquid foods.

Chinese liquor has been consumed by billions of people for over three thousand years. While alcohol consumption is associated with various health risks, it is important to highlight the potential risks of cyanide poisoning in Chinese liquor. Our study revealed significant variation in cyanide levels among different aroma types of liquor, with the highest concentrations observed in the sauce-flavor. Based on the significant variation in cyanide levels among aroma types, we recommend enhancing process control during fermentation and distillation—particularly for the sauce flavor. Monitoring cyanogenic precursors in raw materials could further mitigate risks, though this requires future cultivar-screening studies.

## Figures and Tables

**Table 1:** Effect of different temperatures on linear correlation coefficients.

Temperature	Slope	Intercept	R^2^
20 °C	0.00577 ± 0.00151	0.33518 ± 0.17626	0.78458
40 °C	0.00598 ± 0.00084	0.15828 ± 0.09844	0.92600
60 °C	0.00453 ± 0.00007	0.00979 ± 0.00835	0.99900
80 °C	0.00271 ± 0.00044	0.00649 ± 0.05080	0.90640

**Table 2:** Results of spike recovering experiment.

Background Value (μg/L)	Spiked Value (μg/L)	Average (μg/L)	Spike Recovery Rate
9.246	10	18.722	94.76%
	20	27.552	91.53%
	40	48.640	98.49%

**Table 3:** Results of the precision experiment.

Scheme	Measured Value (μg/L)						Average (μg/L)	Precision
1	47.735	47.810	47.633	47.537	47.522	49.714	47.992	1.18%
2	22.311	22.497	21.983	21.870	22.752	22.533	22.324	1.53%
3	34.326	33.980	34.418	35.273	34.961	35.101	34.677	1.47%

## Data Availability

Data for this article (experimental data and standard curves of cyanide determination) are available at https://doi.org/10.57760/sciencedb.21427. The other original contributions presented in the study are included in the article, further inquiries can be directed to the corresponding author.
